# Trusted Autonomy and Cognitive Cyber Symbiosis: Open Challenges

**DOI:** 10.1007/s12559-015-9365-5

**Published:** 2015-12-23

**Authors:** Hussein A. Abbass, Eleni Petraki, Kathryn Merrick, John Harvey, Michael Barlow

**Affiliations:** School of Engineering and Information Technology, University of New South Wales, Canberra, ACT 2600 Australia; Faculty of Arts and Design, University of Canberra, Canberra, Australia

**Keywords:** Trust, Autonomy, Trusted Autonomy, Trusted Autonomous System, Human–machine teaming, Cognitive Cyber Symbiosis

## Abstract

This paper considers two emerging interdisciplinary, but related topics that are likely to create tipping points in advancing the engineering and science areas. Trusted Autonomy (TA) is a field of research that focuses on understanding and designing the interaction space between two entities each of which exhibits a level of autonomy. These entities can be humans, machines, or a mix of the two. Cognitive Cyber Symbiosis (CoCyS) is a cloud that uses humans and machines for decision-making. In CoCyS, human–machine teams are viewed as a network with each node comprising humans (as computational machines) or computers. CoCyS focuses on the architecture and interface of a Trusted Autonomous System. This paper examines these two concepts and seeks to remove ambiguity by introducing formal definitions for these concepts. It then discusses open challenges for TA and CoCyS, that is, whether a team made of humans and machines can work in fluid, seamless harmony.

## Introduction

We now live in a world surrounded by technology. For some, as implants complement or replace bodily functions, technology also resides within. This new world not only creates opportunities, but also imposes heavy technological challenges. This paper considers two specific technologies: Trusted Autonomy (TA) and Cognitive Cyber Symbiosis (CoCyS).

Trust is a subject that has received tremendous attention from scientists. Social scientists, psychologists, and linguists have studied trust for decades in human-to-human relationships. Further, computer scientists and engineers have realised that automation is unusable if untrusted by humans. Human factor studies have examined interactions between humans and machines, humans and automation, humans and computers, and humans and robotics to explore the role of trust and improve the performance of agents during interactions. These studies have revealed the many elements trust requires.

Presently, the mechanisation of trust within automation systems does not reflect how trust is institutionalised within human social systems. Mechanising trust within automation is not a trivial matter. Further, it is a matter that becomes even more complex as we move from automation to autonomy.

Autonomy is a necessary condition of trust. An agent cannot trust if it does not have the free will to act autonomously in and or in relation to its environment and interfaces. Without free will and autonomy, trust can become a diluted concept.

Trusted Autonomous Systems (TASs) have had a significant and positive impact on society in a variety of areas [1], including in assisting the elderly, medical domains (e.g. surgical robots and rehabilitation), systems that improve robot understanding of human emotions, the use of robots as teachers, space applications, and in entertainment and manufacturing. Trust offers opportunities for autonomous systems; however, without trust, benefits offered by autonomy are severely limited, particularly in complex socio-technical systems where high levels of cooperation are needed.

TA research seeks to design autonomous systems that can be trusted by humans and other autonomous systems. This research begins with the self-evident proposal that there must be a mutual understanding of trust between the truster and trustee. If two parties do not understand the concept of trust, the relationship cannot strictly be defined as a trusting relationship. Thus, autonomous systems cannot be trusted unless they have their own understanding and concept of trust. This premise relies on a distinction between trust and confidence; for example, if a human relies on a machine that does not understand trust, the human only has confidence in the performance of the machine. However, if a machine were able to interpret and understand trust, then a human could trust the machine. Presently, if a machine fails, humans do not strictly view it as untrustworthy, as machines have no free will; rather, the producers or manufacturers of the machine are viewed as untrustworthy for providing an unreliable machine. In these circumstances, an interaction between a machine and a human is no different to an interaction between two humans. However, we acknowledge that this distinction between our position on trust and some literature on the topic is only a guiding (and not a constitutive) principle, and other researchers may consider these examples to represent different forms of trust.

We commence with the simple idea that trust occurs at the interface or in the space of interaction between two or more parties. This interface plays a pivotal role in the formation and deformation of trust. However, to date, this interface has not received sufficient attention by researchers. Most studies evaluate trust while considering one or more pre-designed interfaces. Further, in many studies, the concept of an interface has been limited to a specific form such as a graphical user interface or some other mode of communication. In this paper, the interface represents the integration interface or the space present between two or more parties.

The space interface of interaction among trusting parties forms a web that connects parties according to different relationships. CoCyS is a caricature of the space of interaction between the trusting parties and the associated network, where each node represents an agent and each link defines an interface between two agents within a particular context.

Section 2 of this paper reviews the concepts of trust, autonomy, and TA and introduces definitions to eliminate any ambiguity within the context of this work. Then, the concept of CoCyS is introduced in Sect. 3. Following this, the open challenges in TA and CoCyS are examined in Sect. 4, future work in Sect. 5, and, finally, Sect. 6 concludes the paper.

## Trusted Autonomy

### The Origin of Trusted Autonomy

Huhns and Buell [2] first coined the term TA in 2002 in the context of Internet agents. They equated ‘trustworthy systems’ with TA and structured their argument by first contextualising autonomy within the agent’s literature and then describing the ingredients for trust. Approaching the topic from an agent’s design perspective, they attempted to focus on social autonomy. They noted that agents are sociable and aware of their colleagues and viewed external interactions and coordination with other agents in the environment as constraints on the autonomy of an agent.

Huhns and Buell also discussed and identified the following three ingredients of ‘systemic trust’: understanding, interaction management, and philosophy and societal conventions. These three ingredients have been reformulated as the ability to represent systems using a high level of abstraction to allow systems to understand each other, the ability to manage interactions at the application level, and the transparency of an agent’s ethics and philosophy. They also noted two challenges: credibility and reputation. Credibility is a function of the information provided by a source (i.e. whether the information is credible) and reputation is a function of the source (i.e. whether the source is reputable).

The previous view on TA was limited to Internet agents and included the biased view that trust was a computable concept. In the next section, we consider two approaches to remove these limitations. First, we consider trust and present a generalised model of trust based on objective and subjective dimensions of trust. Second, we provide a definition for TA that can be used in human–human, human–machine, and machine–machine interactions. We then discuss the open challenges for TA research.

### Generalising Trust

#### Human–Human Trust

Trust is a social phenomenon that underpins interactions between two or more human agents. The following social definition of trust was developed for this study:

##### **Definition 1**

Trust is a social contract between two agents. The truster delegates a task to the trustee, but assumes the risk that the trustee might be untrustworthy. The trustee accepts the task, implicitly or explicitly promising to be trustworthy.

Robinson and Morrison [3] discussed the expectation that the truster (T) expects that the trustee (U) will be trustworthy. Thus, the truster’s decision of whether or not to trust the trustee depends on the truster’s level of risk propensity. This dimension of risk offers another perspective on trust as follows:

##### **Definition 2**

Trust is a behavioural attribute dependent on the risk propensity of an agent towards another agent.

Psychologists view trust through a lens of risk; a trusting situation is normally perceived as an ambiguous path [4, 5]. The relationship between trust and risk is common theme within the literature [6–8]. Humans may decide to trust others as they seek to reduce negative risk or increase positive risk (i.e. create opportunities). Thus, in situations that require trust, a judgement needs to be made. Humans make judgements based on an integration of their experiences over time. A trusting decision combines an individual’s judgement and perception of risk with the individual’s experience and cognitive attributes. Thus:

##### **Definition 3**

Trust involves the judgement of a truster agent in relation to a trustee agent based on an integration of the truster’s cognitive attributes and life experience, including, but not limited to, the truster’s experience with the trustee.

Trust exposes trusters to unwanted uncertainties; however, the interdependency that exists between trusters and trustees creates social ties that establish social systems [9]. Trust creates uncertain expectations [10] for trusters. To date, studies have focused on the concept of trust in social and human systems [5, 9, 11], the implications of trust and mistrust [7, 12, 13], and the ethics of trust and antitrust [14]. Lencioni [15] identified five reasons for dysfunction within human teams: absence of trust, fear of conflict, lack of commitment, avoidance of accountability, and inattention to results.

Similarly, sociologists believe that trust allows an operator to manage complexity within a social system [9, 16]. Trust (as a social operator) has a significant impact on all types of interactions across many domains, including marketing [17] and organisations [13, 18–20]. Further, trust is a life-saving phenomenon in areas such as medicine, the military, the police, and safety critical systems (e.g. for airline pilots and operators of nuclear reactors). Thus:

##### **Definition 4**

Trust is a social operator that balances the complexity inherent in social systems and the environment.

Thus far, this paper has highlighted four dimensions of trust that can be grouped as the external states of an agent, that is, behavioural, mental, organisational, and social. These dimensions are referred to as external states because they are subject to one agent’s judgement of the trustworthiness of another agent. A cumulative experience is formed by an agent’s perception of another agent’s behaviour or how that agent thinks, the social system and the organisation. This cumulative experience impacts the judgements made by a truster in relation to a trustee in human–human relationships.

Trust can be seen as a game, whereby the concept of trust requires the delegation from the truster agent (*T*) to the trustee agent (*U*). This delegation is associated with the level of risk (*R*) that the truster agent will be exposed to if the trustee fails and the level of reward or gain (*G*) that truster will receive if the trustee succeeds. Without loss of generality, trust can be modelled as a two-player social dilemma game [21]. Abbass et al. [22] provided a generalisation to an *N*-Player.

**Table:** 

Player 1	Trust		Do not trust
Player 2	Reciprocate	Defect	**0**, **0**
Utility	**G**, **G**	$-\mathbf{1}$, **1**	

With the following constraint: $0 < G < 1$. Thus, gain acts as the reward when the truster trusts the trustee and as an opportunity loss when the truster does not trust a trustworthy trustee. The two numbers in each cell represent the reward received by the truster and trustee, respectively, given a particular pair of actions.

There are two types of temptation. First, the temptation of the truster to trust, (*M*1) and, second, the temptation of the trustee to be untrustworthy (*M*2), where:

$$\begin{aligned}&M1 = G\\&M2 = 1 - G \end{aligned}$$

The above game definition is very comprehensive, but is disadvantaged, as the two types of temptation are dependent on the gain (i.e. *G*) and one temptation cannot be changed without changing the other. To generalise the game, it was redefined as:

**Table:** 

Player 1	Trust		Do not trust
Player 2	Reciprocate	Defect	**0, 0**
Utility	**G**, **Z**	−**R**, **W**	

With the following constraint: $G > 0$ and $W > Z$. The game is a social dilemma when: $G + Z > W$.

The two types of temptations can then be defined as:

$$\begin{aligned}&M1 = G \\&M2 = W - Z \end{aligned}$$

The revised game explains many of the definitions and statements of trust discussed in the literature. It shows why trust requires acceptance of vulnerability [23] (i.e. because the truster relies on the trustee to gain but the trustee can defect and cause the truster to suffer loss). Mayer et al. [23] stated that a truster’s decision must also be based on free will if the decision is to be deemed a trusting decision.

#### Human–Machine Trust

Similar to social systems, human–machine trust can enhance performance in complex situations. If a human trusts a trustworthy machine, the human may delegate tasks to the machine to assist him/her to make decisions and complete tasks in complex situations. Further, if this trust is positively reinforced, task performance improves. Thus, the effect of trust on relationships and interactions between human and machines is very similar to the effect of trust between humans in organisations or social systems. However, two questions arise: Does the concept of trust mean the same thing in human–machine relationships and human–human relationships? In interacting with machines, are the strategies humans use similar to those they use when interacting with other humans?

Barber [6] developed a taxonomy of trust centred on three concepts: persistence, technical competency, and fiduciary obligations. Persistence allows for understanding and the creation of mental models of physical processes that can be used to predict future events. Technical competency can be categorised as: expert knowledge (i.e. knowledge-based behaviours), technical facility (i.e. rule-based behaviours), and routine performance (i.e. skill-based behaviours). Fiduciary obligations refer to a truster’s forced reliance on a trustee to perform his/her moral obligations due to an inability on the truster’s behalf to evaluate the trustee’s technical competency .

Muir [24] used a combination of elements from the work of Barber [6] and Rempel et al. [25] to form the following formal definition of trust that applies across human–human and human–machine contexts:

1 $$T_{ij} = [ E_{i(P_n+P_m)} ] + [E_{i{\mathrm{TCP}}_j}] + [E_{i{\mathrm{FR}}_j}]$$

where *T* is trust, *i* is the truster, *j* is the trustee, *E* is expectation, *P* is persistence, *n* is natural orders, *m* is moral social orders, TCP is technically competent performance, FR is fiduciary responsibility.

Muir [24] outlined four ways to improve the human–machine trust calibration and identified appropriate values for the parameters of the model to accurately assess an appropriate level of trust. First, Muir noted that a user’s ability to perceive a decision agent’s trustworthiness should be enhanced. He outlined a number of ways to achieve this improvement, including training to better understand how automation works, providing the user with explicit predictability data on the automation’s competency and responsibilities, offering the user the means to receive intention information from the automated system, and finally, allowing the user ample chances to interact with the automation to develop calibrated expectations.

Second, Muir asserted that the user should be allowed flexibility to modify his/her criterion of trustworthiness (a complementary concept to the truster’s calibration of trust except that this is operated by the trustee). Flexibility can be achieved through a transparent automation in which expected competency, a history of competence and responsibility, is communicated to the user. The criterion level of acceptable performance should also be transparent.

Third, Muir argued that users should have the ability to allocate functions within the system instead of being forced to operate a rigid system. By placing the human in control of the machine, the responsibility and authority for making decisions are given to the human and thus the humans feel in control.

Fourth, Muir noted that poor calibrations of trust may cause inaccurate expectations of persistence, competence, and/or responsibility. Thus, it is important to identify and select any poorly calibrated dimensions of trust.

The model proposed by Muir [24] led to what is known in automation as ‘calibrated trust’. In this model, trust is calibrated along the above four dimensions.

Mayer et al. [23] proposed three general bases of trust: ability, integrity, and benevolence. Ability refers to the degree of the trustee’s ability to perform a task. Integrity is the degree by which the trustee’s actions match the values of the truster. Benevolence refers to the degree by which the trustee’s actions match the goals and motivations of the truster.

Jian et al. [26] used a three-phase study to understand and measure trust between humans and automation. In the first phase, researchers conducted a three-condition experiment to collect words related to trust and distrust. In the second phase, they used a questionnaire to measure the proximity of these words to one another. Finally, in the third phase, they asked participants to rate different pairs of words. Their results showed that trust and distrust are two opposite concepts, and that people’s perceptions of trust did not change when general trust was compared across human–human and human–machine relationships.

In another study, Dzindolet et al. [27] found that trust impacts automation reliance decisions. An important matrix can be derived from their work, that is, the relationship between trust and reliance as depicted in the following Table 1.

**Table 1:** Relationship between trust and reliance

Reliability	Trust	Distrust
Automation > manual	Automation reliance	Disuse
Automation < manual	Misuse	Self-reliance

An important component of Dzindolet et al.’s [27] research relates to the results they found in different experiments. Specifically, they found that participants with no experience of a particular automated aid tended to have a positive bias to deem the aid as trustworthy; however, this also led to misuse when the automated aid was not as good as their own self-judgement. Further, when participants were allowed to interact with the automated aid to gain experience, they tended to disuse the automated aid. Interaction and experience caused participants to reduce their trust levels with the machine even in cases where the machine’s performance was superior to the participants. When participants were given continuous feedback on the relative performance of the automated aid (as compared to their own performance), however, even without the exact decision of the automated aid being revealed, disuse was eliminated. Thus, Dzindolet et al. concluded that offering humans feedback on the performance of an autonomous system (when it is performing better than the human) led to humans learning when to rely on automation, and this increased humans’ trust level in the automation.

Marsh and Dibben [28] proposed three layers of trust:

- *Dispositional trust* similar to what Dzindolet et al. [27] found, most individuals tend to trust automation;
- *Situational trust* the context (i.e. environment) influences the truster’s ability to trust the trustee. Similar to the findings of Dzindolet et al. [27], an operator’s interactions with the system cause contextual variations in the operator’s mental state.
- *Learned trust* where previous experiences (i.e. interactions with the same or similar trustees) influence the truster’s ability to trust the trustee.

In a human–machine teaming exercise, Sycara and Lewis [29] identified the communication of human intent as the greatest obstacle to achieving effective human–machine teams. They noted that the same three factors are found in human–machine teams and human–human teams: mutual predictability among team members, shared understanding, and the ability of team members to adapt to one another.

In a subsequent study, Sycara and Suktghankar [30] identified four dimensions of effective human–machine teaming: information exchange, communication, supporting behaviour, and team initiative/leadership.

Joe et al. [31] evaluated automation in relation to teams and found the following eight issues between human–human teamwork and human–machine teamwork: challenges building a shared understanding of contexts, difficulties anticipating and predicting intentions at individual and team levels, machines’ inability to adapt at the same rate as humans, an inverse relationship in the amount of interaction between humans and machines with automation levels, disruptions caused to human teams when automation is introduced, the effects of increased workloads on humans, and poor communication protocols between humans and machines.

Joe et al. [31] also identified seven key principles for effective teamwork using nuclear power plant crews and used these principles to evaluate human–machine teamwork. The seven key principles were as follows: belief in the concept of a team, effective communication, team leadership (normally by a human, but this may change in the future), monitoring individual and group performance and providing feedback, coordination and assistance, awareness of internal and external performance shaping factors that affect teams' processes and awareness that each individual’s mental model is unique, and that it is difficult to create shaped mental models in teams.

**Fig. 1:**
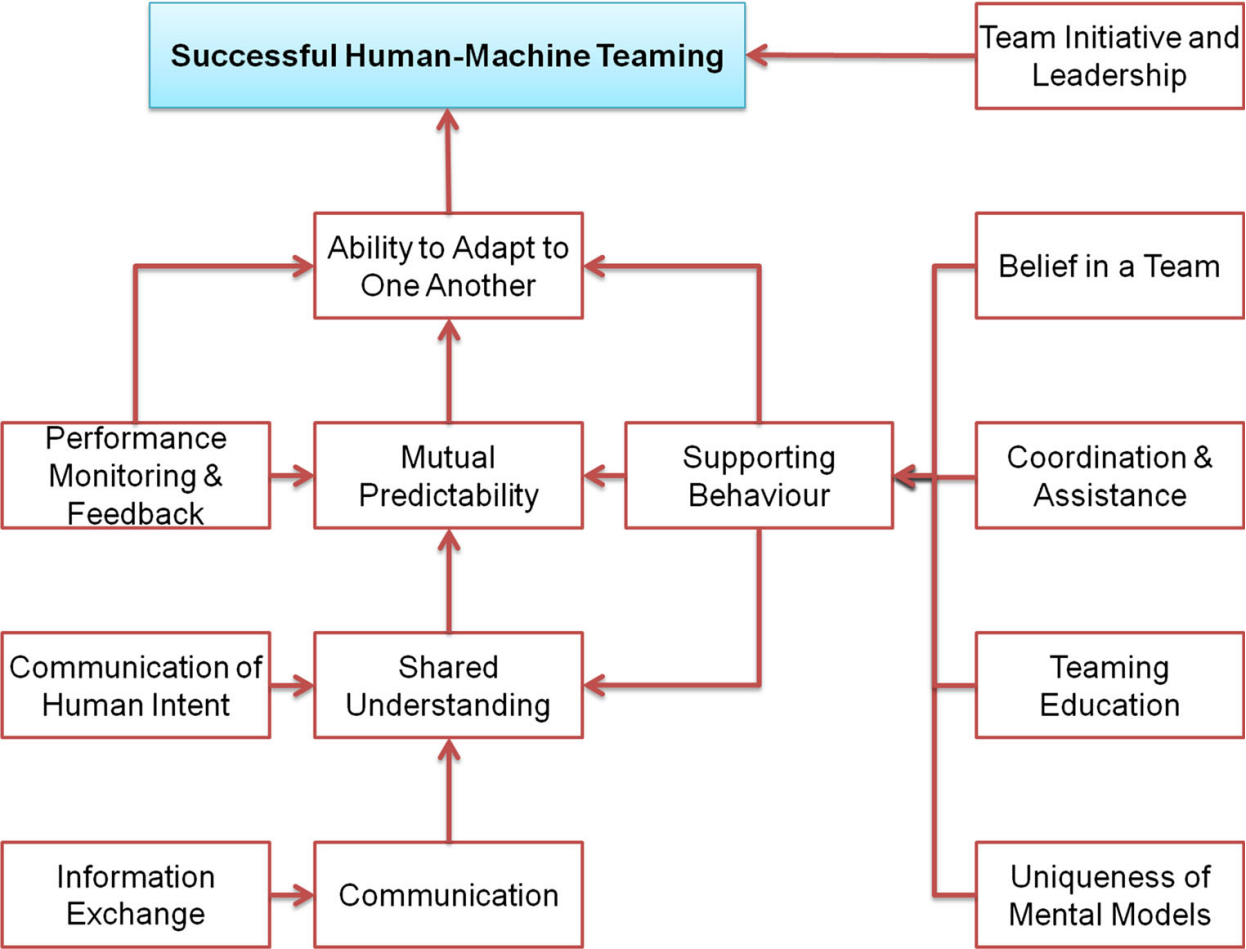
A model for successful human–machine teaming

Figure 1 sets out a simple model of successful human–machine teamwork that captures the information discussed above. This model connects supporting behaviours with the behaviours required for successful human–machine teaming.

Hoff and Bashir [32] systematically reviewed empirical automation research on factors influencing trust in automation and then proposed a comprehensive model that incorporated these factors. Their model covered two phases: prior to interaction and during interaction. Prior to an interaction, three factors influence decisions to trust; dispositional (i.e. culture, age, gender, and personality traits), situational (i.e. internal and external variability), and initial learnt trust (i.e. preexisting knowledge). During interactions, trust is dynamically learnt through reliance on the system, and it is a system’s design features and performance that impact trust.

Camp [33] operationalised trust using three concepts: privacy, security, and reliability. Camp defined privacy as the right to autonomy, seclusion and data as property and differentiated between security and privacy by positioning security as a means to control digital information. Conversely, privacy demands that people are able to control their personal information. Camp also discussed the sometimes neglected issue that security can conflict with reliability; for example, a highly secure system may be so sensitive to the need for a large amount of user data for authentication purposes that it is perceived as unreliable by users.

**Fig. 2:**
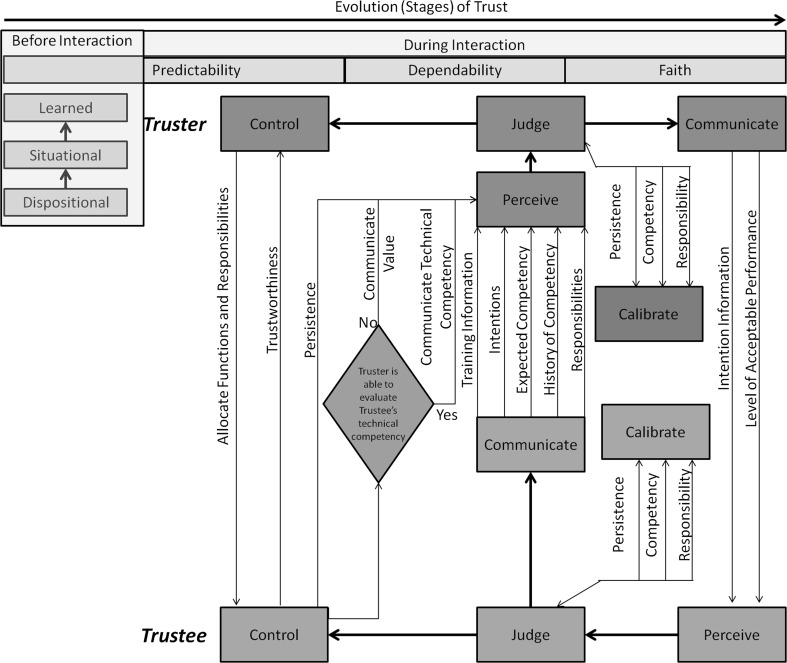
A summary of the literature review on trust

Figure 2 summarises the above literature into a comprehensive model on the dynamics of trust. Both the truster and trustee must engage in the processes of perceiving, communicating, exercising control on one another, making internal judgements as to how to integrate perceived information and deciding what actions to produce. The truster calibrates the trustee’s level of trustworthiness, and the trustee calibrates its ability to influence and shape the truster’s perception of its level of trustworthiness.

**Fig. 3:**
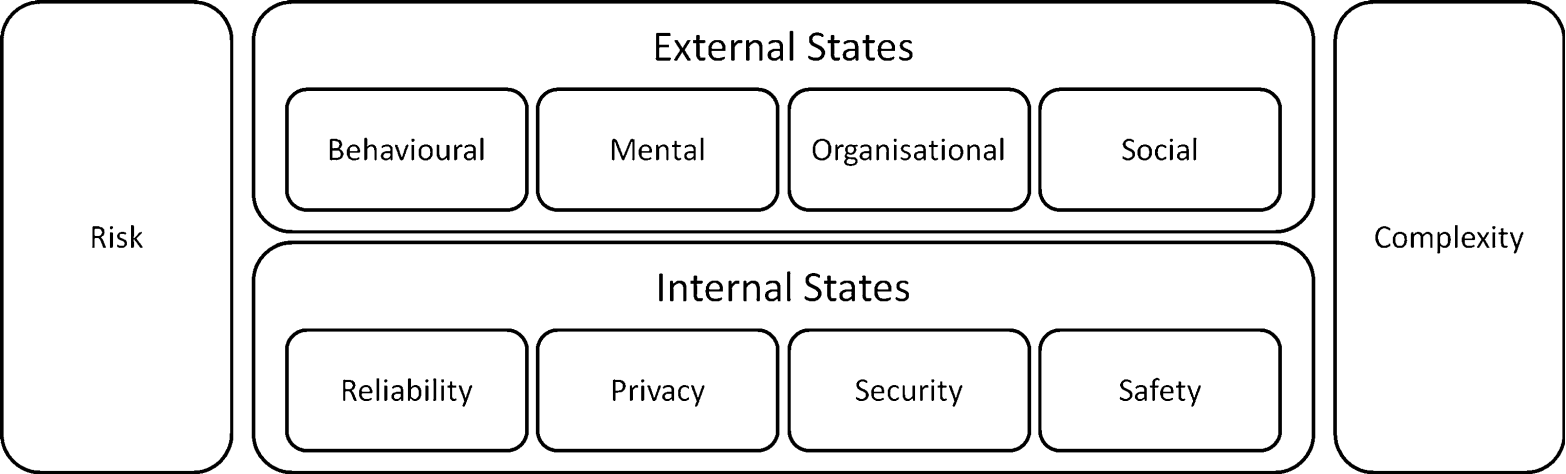
An overall encompassing trust model for humans and machines

Figure 3 summarises the factors related to humans and machines and brings together relevant internal and external elements of trust. It emphasises the two approaches taken to trust: the risk-taking role (that psychology studies have emphasised) and the complexity management role (that sociology studies have emphasised). Elements of trust are grouped into internal and external elements. The external elements are associated with those complex elements that define and shape trust in humans: behavioural and mental attributes of an agent and organisation and the social attributes of the society. The internal elements are associated with computable dimensions, including reliability, privacy, security, and safety.

#### Doubts and the Two-Way Nature of Trust

As stated above, Jian et al. [26] showed that trust and distrust are two opposite concepts. A doubt is a trigger for distrust. The concept of doubt can act as a trigger for a vicious cycle that promotes distrust if the truster doubts the trustee’s ability to perform a task or doubts the privacy and security of the communication channel between the truster and the trustee or if the trustee doubts that the truster is genuine.

Doubts are ‘uncertainty’ viruses in both human social systems and human–machine social systems. Once a doubt exists, it takes a long time and evidence for it to be eliminated. It is contended that humans are worse than machines at eliminating doubts because of confirmation bias. Once a human doubts another human or a machine, the former will interpret the actions of the latter with this doubt in mind, a bias that tends to confirm doubts. Consequently, humans take a longer time than machines to eliminate their doubts and thus trust again.

Trust in many situations is a two-way interaction. The truster needs to trust that the trustee will not disappoint their expectations. The trustee also needs to trust that the truster is not tricking the trustee or wasting the trustee’s time. This two-way flow of trust between the truster and the trustee should be split into two different relationship types to remove ambiguity.

By way of example, assume that a truster entrusts a trustee with a delegated task. If the trustee perceives this task to be a trick by the truster to occupy the trustee’s time or weaken the trustee, the trustee may not accept the task or may accept the task with doubts. In this example, the two-way flow model has two one-way flows; in the first flow, the truster trusts the trustee with the task, and in the second flow, the trustee becomes the truster and must trust the truster with his/her time and effort. Thus, we have two different types of trust in this situation that need to be modelled and handled independently even though the decision of either party may impact the decision of the other.

Distrust is linguistically antithetical to trust; however, the two concepts of trust and distrust have been distilled and contrasted. Lewicki et al. [34] argued that despite being independent constructs with different sets of expectations, trust and distrust can coexist; for example, someone can trust another individual for a particular purpose, but also have feelings of distrust towards that person for other purposes. In the work of Lewicki et al. [34], trust and distrust entailed different expectations and occupied elements on a continuum. They hypothesised four different relationships between trust and distrust: (1) low trust/low distrust; (2) high trust/low distrust; (3) low trust/high distrust; and (4) high trust/high distrust.

Saunders et al. [35] examined expectations and categorisations in definitions of trust and distrust and contended that any definition of trust must include cognitive and affective elements, and that these two different concepts can be present at the same time. They concluded that trust is a multifaceted phenomenon, highly dependent on context. We rewrite Eq. 1 to accommodate context as follows:

2 $$T_{ij} = f_c([ E_{i(P_n+P_m)} ] + [E_{i{\mathrm{TCP}}_j}] + [E_{i{\mathrm{FR}}_j}])$$

where $f_c$ is an operator that manipulates trust based on some contexts, *c*.

Other work on trust and suspicion [36] examined the impact of trust on motivational tendencies. Deutsch [36] hypothesised that trusting behaviour may have positive or negative motivational consequences depending on whether the trust is fulfilled. However, if the fulfilment of trust is not certain, individuals are exposed to conflicting tendencies to engage in suspicion or avoid trusting behaviour in future.

#### Levels of Trust

If trust is a binary concept, then the question arises: Do decisions to trust or not trust occur on a scale? Table 2 defines different levels of trust from the truster’s perspective. A truster makes two different judgements; the first relates to the truster’s belief of the level of trust, and the second is based on the truster’s belief as to the trustee’s level of trustworthiness. A truster may evaluate a trustee’s level of trustworthiness as very high, but evaluate the transaction as a medium trusting decision; for example, a truster may believe that a trustee is very trustworthy and delegate a low-risk task to the trustee. In this example, the truster’s level of trust in the decision is low, despite the truster’s evaluation of the trustee’s level of trustworthiness as being very high.

**Table 2:** Truster’s judgement on levels of trust and trustworthiness

Trustee’s temptation to defect	Truster’s temptation to invest		Truster’s judgement on level of
	Low	High	
Low	Low	Medium	Trust
	Low	High	Trustworthiness
High	High	Very high	Trust
	Medium	Very high	Trustworthiness

Table 2 shows the levels of trust and trustworthiness across two dimensions (i.e. the truster’s temptation to invest and the trustee’s temptation to defect). These two dimensions describe different aspects of trusting decisions.

In circumstances where a truster gains a lot by trusting, the truster’s temptation to trust is very high. Additionally, if the trustee’s temptation to defect is also very high, if the trustee does not defect, trustworthiness is very high. However, if, in these circumstances, a truster makes a decision to trust, the level of trust needs to be high, as the truster assumes the risk that the trustee may defect.

### Autonomy

The words ‘autonomous’ and ‘autonomy’ originate from the Greek words $\alpha \upsilon \tau o \nu o \mu o \varsigma$ and $\alpha \upsilon \tau o \nu o \mu \iota \alpha$, respectively. The Greek origin consists of two words $\varepsilon \alpha \upsilon \tau o \varsigma,$ meaning ‘self’, and $\nu o \mu o \varsigma,$ meaning ‘law’. Thus, ‘autonomy’ comprises aspects of self-governance and suggests that agents rely on their own laws and work independently.

Before discussing autonomy further, we examine different levels of automation and intelligence, two concepts that must be considered in any discussion of an autonomous system. Automation can be defined as ‘technology that actively selects data, transforms information, makes decisions, or controls processes’ [37].

**Fig. 4:**
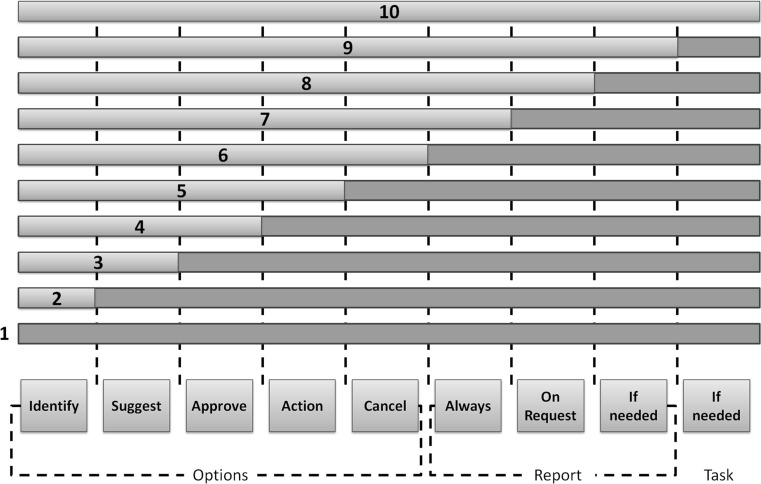
Levels of automation adopted from Sheridan and Verplank [39]

Sheridan [38] proposed 10 degrees of automation (see Fig. 4). The lowest degree being ‘the computer offers no assistance: humans must do it all’, and the highest degree being ‘the computer selects and executes the task, and decides whether it should tell the human or not’.

Digney et al. [40] defined seven levels of intelligence in relation to vehicles; however, these levels are generalisable and can be applied to machine intelligence generally. The seven levels of intelligence are as follows:

- *Level 1* Clever handcrafted algorithms without adaptation in structure or parameter.
- *Level 2* Handcrafted algorithms adapted by hand and distributed to the automation through the network.
- *Level 3* Handcrafted algorithms tuned automatically across the network.
- *Level 4* Adaptation algorithms that modify parameters online.
- *Level 5* Adaptation algorithms that modify structures online.
- *Level 6* Discovery and exploitation of useful relationships.
- *Level 7* Creative extrapolations for predicting relationships.

Digney et al. also listed the following enabling research areas to allow for machine intelligence: self-defining representations and control structures; reduction in world to functional abstractions; a unified approach to combined symbolic and real-valued representations; a unified approach to learning, planning, and abstraction; collective control and learning for teams of vehicles and agents.

Freed et al. [41] distinguished between automation (i.e. time-based commands) and advanced automation (i.e. goal-based commands) and defines a third term: ‘variable autonomy’ whereby intelligent control software adapts the degree of automation. Autonomy can be varied by altering the complexity of commands, the resources consumed during operation, the number of subsystems under control, the allocation of responsibilities, the circumstances in which the system can override or allow manual operation, and the circumstances in which the system can request user information.

Freed et al. argued that on-board automation (as opposed to remote or ground-based automation) offers advantages such as freeing up communication infrastructures, removing communication delays and restrictions and provides possibilities for on-board automation to replace crews in the performance of some actions. However, such automation can impose challenges (e.g. in relation to software verification and validation and the correct specification of domain knowledge), especially for infrequently used software.

Huhns and Buell [2] viewed interaction as a constraint on autonomy and claimed that coordination activities among agents constrained the autonomy of each agent. They effectively argued that an interaction between two agents constrains the freedom (i.e. free will) of each agent. However, we did not adopt this approach, as freedom should be defined within a social system (whether it be a system of humans and/or machines) in which two or more agents interact to improve the overall performance of the system, rather than the individual agents. Further, autonomy cannot be defined as the ability of one agent to act without the constraints of other agents. Thus, in this study, autonomy was defined as:

#### **Definition 5**

Autonomy is the freedom to make decisions subject to, and sometimes in spite of, environmental constraints according to the internal laws and values that govern the autonomous agent.

### On Trust and Autonomy

Trust requires a certain level of autonomy without which the situation cannot be trusting. An agent with no free-will may be able to make decisions on trust, but cannot be autonomous.

**Fig. 5:**
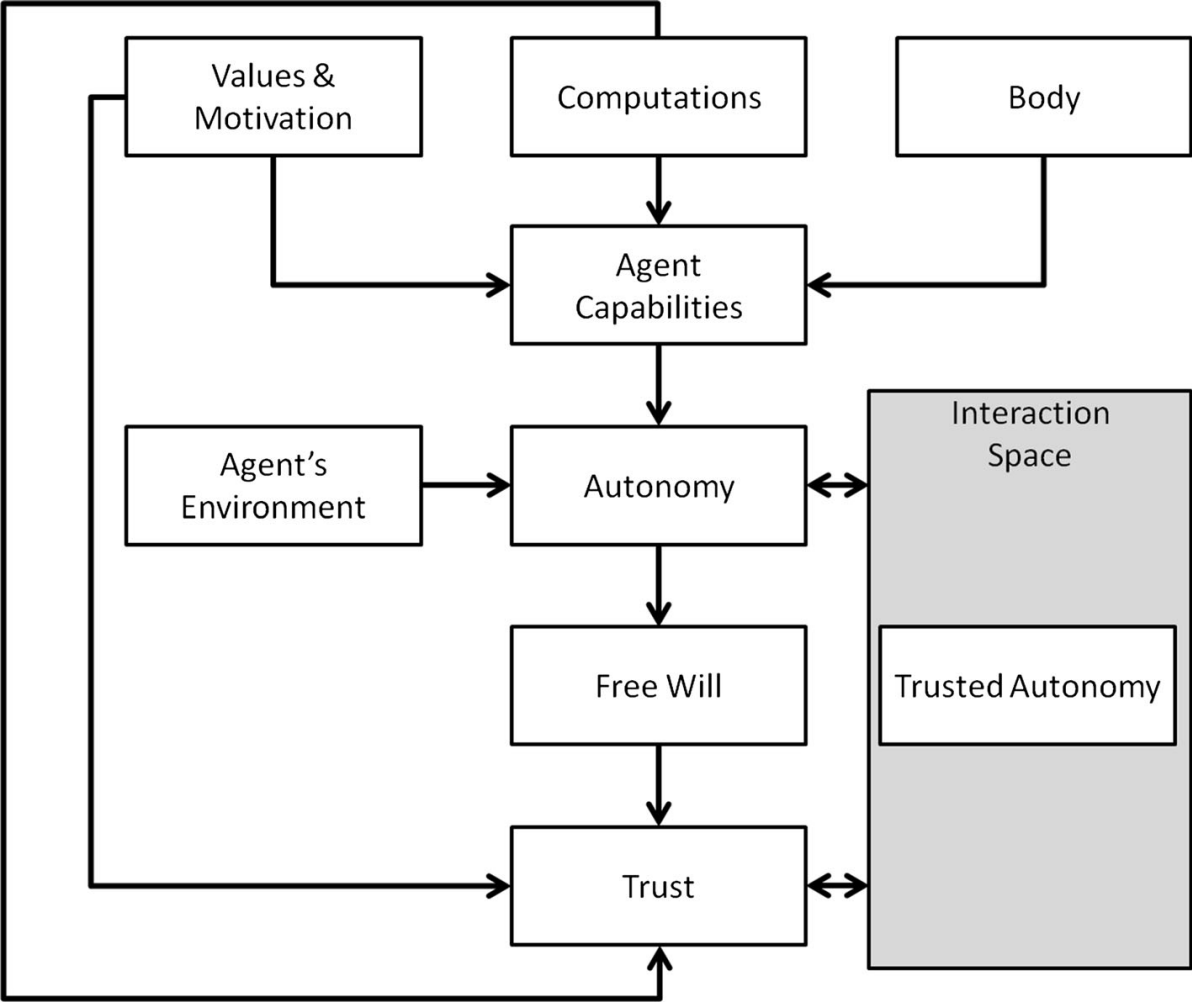
Relationship between trust and autonomy

Figure 5 captures the relationship between trust and autonomy. It emphasises that autonomy depends on an agent’s capabilities and the environment within which that agent exits. An agent’s capabilities include the body, software (i.e. control, decision-making, or the brain of an agent) and the agent’s value system and motivational derivers. Our previous work on body–brain coevolution [42, 43] showed a trade-off exists between the constraints an agent’s body form and the level of motion that an agent can exhibit. An agent may have the potential to be fully autonomous, but the environment may constrain its behaviour and thus deny it its autonomy; for example, an autonomous car cannot exercise its autonomy if it is driven into an ocean.

As autonomy implies free will, trusting decisions require free will. TA occurs in the space that resides between agents, that is, the space of interaction.

#### **Definition 6**

TA occurs in the space of interaction residing between agents.

### The Role of Motivation for Trust and Autonomy

Motivation is a core concept in human decision-making. A number of studies have shown that differences in motivation effect trust and autonomous decisions. Thus, in studying trust and autonomy, motivation in human systems should be considered in the design of computational motivation models. These models must then be integrated with an autonomous system.

#### Implicit Motives

Enduring motive dispositions or ‘implicit motives’ [44] are preferences for certain types of incentive acquired in early childhood [45]. Incentives are the situational characteristics associated with the possible satisfaction of a motive. Incentives are either implicit or explicit. Examples of implicit incentives include challenges to personal control in a performance situation (i.e. an achievement motive), opportunities for social control (i.e. a power motive), and opportunities for social closeness (i.e. an affiliation motive). In humans, differences in implicit motives have also been linked to differences in preferences for explicit incentives such as money, points or ‘payoffs’ in a game [46–49] or during other types of strategic interaction [50]. Table 3 summarises some of the salient characteristics associated with each motive.

**Table 3:** Characteristics that may be observed in individuals with a given dominant motive [44, 45, 51]

Dominant motive	Possible characteristics
Achievement	Prefers moderately challenging goals
	Willing to take calculated risks
	Likes regular feedback
	Often likes to work alone
Power	Wants to control and influence others
	Likes to win
	Likes competition
	Likes status and recognition
Affiliation	Wants to belong to a group
	Wants to be liked
	Prefers collaboration over competition
	Does not like high risk or uncertainty

#### Achievement Motivation

Achievement motivation [52] drives humans to strive for excellence by improving personal and societal standards of performance. A number of achievement models exist, including Atkinson’s risk-taking model (RTM) [53]. More recently, achievement motivation has been examined from an approach–avoidance perspective [54]. An interesting aspect of achievement motivation in relation to risk and trust is the hypothesis that success-motivated individuals perceive an inverse linear relationship between incentives and the probability of success [50, 55] and tend to favour goals or actions with moderate incentives, a moderate probability of success, calculated risks, and of moderate difficulty. Such individuals are often content to work alone to achieve these goals.

Approach–avoidance motivation has also been studied in the social domain [56] to model the differences in goals directed towards positive social outcomes (such as affiliation and intimacy) and those directed towards negative social outcomes (such as rejection and conflict) [57]. The idea of approach–avoidance motivation, together with the concepts of incentive and probability of success, is particularly important not only in achievement motivation, but also in power, affiliation, and other forms of motivation [45].

#### Power Motivation

Power is a domain-specific relationship between two individuals, characterised by an asymmetric distribution of social competence, access to resources or social status [45]. Power manifests in unilateral behavioural control and can occur in a number of different ways. By way of example, consider individuals *A* and *B*:

- Reward power is exerted if *A* is in a position to satisfy one of *B*’s motives and makes such satisfaction contingent on *B*’s behaviour.
- Coercive power is exerted if *A* is in a position to punish one of B’s behaviours by withdrawing *B*’s opportunity to satisfy certain motives and makes this punishment contingent on B’s behaviour;
- Legitimate power is derived from norms internalised by *B* that make *B* aware that *A* is authorised to regulate their behaviour;
- Referent power arises from *B*’s desire to be like *A*;
- Expert power is determined by the extent to which *B* perceives *A* to have special knowledge or skills in a particular area; and
- Informational power is exerted when *A* communicates information to *B* that causes *B* to change his/her beliefs and behaviour.

Five components of fear of power (i.e. inhibition tendencies that lead to avoidance behaviours) have also been identified : fear of the augmentation of one’s power source, fear of the loss of one’s power source, fear of exerting power, fear of the counter-power of others, and fear of one’s power behaviour failing. These inhibition tendencies moderate power by channelling the expression of power into socially acceptable behaviour. Another balancing factor that works in conjunction with power motivation is the affiliation motivation.

#### Affiliation Motivation

Affiliation refers to the class of social interactions used by individuals to seek contact with other, formerly unknown or little known, individuals and maintain contact with those individuals in a manner that both parties experience as satisfying, stimulating, and enriching [45]. The need for affiliation is activated when an individual comes into contact with another unknown or little known individual.

Similar to power motivation, affiliation motivation is thought to comprise two contrasting components: hope of affiliation and fear of rejection. When unfamiliar people interact, the hope component is activated first. Under the influence of affiliation motivation, contact is initiated. As familiarity with the person increases, the closer the relationship between the people becomes and the more painful it would be if rejection occurs. The fear of rejection is activated and becomes increasingly strong. Sensitivity to relevant signals is heightened until the point of maximum conflict between approach and avoidance is reached. When fear becomes dominant, the closeness of the relationship is diminished until the fear motivation decreases and the affiliation motivation dominates once again and the cycle then begins anew.

The maximum approach–avoidance conflict occurs when both components are equally strongly aroused. However, the avoidance tendency is activated later, and the gradient of avoidance is steeper than the gradient of approach. Specific affiliation-related goals include:

- Being in the company of others,
- Cooperating,
- Exchanging information, and
- Being friends, where trust is an important operator.

Individuals high in affiliation motivation may also be intent on effecting reconciliations with others, make more suggestions to change the attitudes of others to bring those attitudes more into line with their own, avoid games of chance, and initiate fewer acts that might cause conflict (this may also mean that they initiate fewer cooperative acts). Individuals with a medium-to-high affiliation motivation may be less deceptive than those with a low affiliation motivation.

The power and affiliation motivation has been the subject of detailed analysis [58, 59]. McClelland [59] further classified affiliation motivation as comprising affiliative and cynical trust and power motivation as comprising agentic and stressed power. Affiliative trust refers to positive expressions of affiliation, such as proposing marriage because of love. Cynical trust refers to negative expressions of affiliation, such as proposing marriage to gain access to another’s money. McClelland [59] found that affiliative trust and a greater sense of agency, as measured by associative thought content, are associated with better health.

McKay [60] developed a coding scheme that assesses the strength of two types of sentiment in affiliative relationships: specifically, trust and mistrust. Individuals who view relationships as enjoyable experiences that turn out well score high on the trust subscale. Conversely, the mistrust subscale assesses expressions of negativity and cynicism about relationships. Scores on the trust and subscales can be considered independently or combined to form a measure of affiliative trust versus mistrust. This coding scheme continues to be used in contemporary research, including the analysis of social relationships between astronauts on an international space station [61].

### Trusted Autonomy Defined

In TA, autonomy is defined in the context of responsible autonomy as follows:

#### **Definition 7**

Responsible autonomy arises from the conscious delegation of a task by one agent to itself or to another agent; however, the agent who is delegated to carry out the task must be known to have the capacity to perform the task and have the autonomy to accept or reject the task.

In the above definition, an agent is allowed to delegate a task to itself. This does not eliminate the need for trust; rather, it generalises the concept of trust to oneself—the agent delegates the task to itself, thus, if it trusts itself to undertake the task and be able to undertake the task.

Responsible autonomy places the responsibility on both parties. The delegator is responsible for ensuring that the delegatee can perform the task; however, the delegatee is responsible for communicating its abilities to the delegator.

Based on the above definition, autonomy is either self-decided or achieved by another machine or human delegating the task. In the latter case, an agent becomes autonomous when a task is delegated to it and the agent is left to decide how to perform that task and what to do when the task is complete. An autonomous agent can decide to delegate the task to itself or to another agent. Thus, the concept of delegation sits at the heart of the concept of autonomy.

Accordingly, a less formal definition of TA is:

#### **Definition 8**

TA refers to an interaction between two or more self-governed autonomous intelligent systems (including humans) in which one party to the interaction is willing to delegate a task that will make it vulnerable to the other party (or parties) and the other party (or parties) is (are) willing to accept and autonomously perform the task.

Delegation can be explicit or implicit; for example, a group of agents cooperating to push a car in real time may hold a shared understanding that the group has delegated the task to the group members (i.e. delegation does not need to be an explicit process/contract).

To avoid any confusion in relation to the concept of autonomy, the term Systemic Autonomy is introduced and defined as:

#### **Definition 9**

Systemic Autonomy is a social contract between two agents (e.g. two humans, two machines, or a human and a machine) or an agent and itself. The first agent (the delegator) delegates a task to a second agent (the delegatee). This contract comprises two sections. The first section describes the level of delegation from the delegator to the delegate, including any constraints in relation to the delegatee delegating sub-tasks to others. The second section describes the ability of the delegatee to perform the delegated task at the level expected and described in the first section, and the delegatee willingness to accept the task.

Systemic Autonomy requires trust, as the decision to delegate may create negative risks for the delegator. Thus, TA was formally defined as:

#### **Definition 10**

TA refers to a situation in which an autonomous agent willingly becomes vulnerable by delegating a task to itself or another autonomous agent.

This definition requires that two conditions be present in any TA decision: first, the delegation makes the delegator vulnerable; second, the delegation is made deliberately with free will. The concept of free will is a necessary condition of trust and, somewhat surprisingly, a necessary condition for autonomy.

The definition also requires that both parties in TA are autonomous agents and that each has a degree of autonomy. The minimum acceptable degree of autonomy in this relationship is the capacity of the delegator to delegate tasks and the capacity of the delegatee to evaluate and accept the delegated tasks and communicate an ability to perform the tasks.

In TA, the delegator becomes a truster and the delegatee a trustee. The level of vulnerability is a measure of propensity to trust. This previous discussion is not limited to one or two agents; a group could represent a single agent in this encapsulation. Thus, the definition of TA was generalised as follows:

#### **Definition 11**

TA is the ability to form teams of humans and/or machines that make educated and conscious decisions to delegate risky tasks among team members seamlessly and symbiotically

#### Trusted Autonomous Agents Need to Influence and Shape Others

Rempel et al. [25] conjectured that trust between humans is a dynamic expectation with predictable changes that begins with predictability before transitioning to dependability and, finally, to faith. Faith provides emotional security to individuals and is thus harder to influence or shape than dependability and predictability. Rempel et al. also demonstrated that reliability, as a basis for predictability, is important in making trust judgements.

Thus, interactions among agents impact each agent’s level of trust for the other agents. Smart agents should be conscious of this interaction and its implications. The question then arises within TA is: Can an autonomous agent be smart enough to influence other agents and shape its own environment so that it becomes trusted?

However, before examining these concepts any further, we will set out definitions for influence and shaping. Larson et al. [62] distinguished between influence and shaping. Shaping is viewed as a change to an organisation or environment. Influence is viewed as fostering the attitudes, behaviours or decisions of individuals or groups. Unlike Larson et al. [62], the majority of the researchers tend to assume that an influencing operation leads to shaping, such that an influence works when it exerts a form of social power. However, Larson et al. [62] noted six sources of power: informational, coercive, reward, legitimacy, expert, and referent.

This paper adopts definitions consistent with Larson et al.’s, but elects to use the more accurate term ‘effect’ (rather than the inappropriate term ‘change’), as in certain circumstances influence and shaping must operate to maintain the status quo; for example, if a trustee agent is attempting to influence a truster agent by changing the truster’s belief about the trustee agent, an agent ‘*C*’ can attempt to counteract agent the trustee agent’s influence by influencing the truster agent to maintain its belief about the trustee agent. Thus, influence does not necessarily require a change to occur, only that an effect is achieved.

##### **Definition 12**

Influence is an action that causes an effect in the attitude or behaviour of an agent.

##### **Definition 13**

Shaping is an action that causes an effect in the environment of an agent.

Coble [63] wrote a thesis examining a decision-making model among nurses. Concepts included in the model were creativity, experience, leadership, education, risk-taking, and informatics. An analysis of the 510 returned questionnaires (that included demographic data and responses to bipolar questions) focused on ascertaining influences on decision-making. A correlation analysis revealed that leadership had the biggest direct effect (0.33), indirect effect (0.19), and total effect (0.52) on decision-making. Experience (0.32), creativity (0.24), and education (0.24) followed in total effect . Finally, risk-taking (0.14) and informatics (0.16) had the smallest direct effects on decision-making. In examining human systems, Coble’s study identified the key dimensions in an autonomous agent that influence trust building.

Servi and Elson [64] introduced a new definition of influence and applied this definition to online contexts such as ‘the capacity to shift the patterns of emotion levels expressed by social media users’. They proposed that measuring influence entails identifying shifts in users’ emotional levels followed by an examination of the extent to which these shifts can be connected to a user. However, a question arises: Whether the process of influence creates a shift in patterns of emotions that can be detected in the short-term? Can the persistent application of the influencing process create a long-term shift and shape the environment as a whole?

Shmueli et al. [65] discussed computational tools that measure processes for shaping and affecting human behaviour in real-life scenarios. Trust was identified as a factor that could influence humans in a social system. They used a case study to introduce a new method of measuring trust and determining its applicability to human behaviour. Their findings suggested that trust can be operationalised and used to predict passive sensing and network analysis. Further, trust was found to have a significant effect on social persuasion. The experiment showed that trust was significantly more effective than the closeness of ties between agents in determining levels of behavioural change.

## Cognitive Cyber Symbiosis

### From Adaptive Automation to Augmented Cognition

The concept of adaptive automation [66] was originally introduced to address the negative effects of static automation. In static automation, a task is completely automated, and the main function of the human operator is to monitor failures in automation. However, studies showed that human performance degrades rapidly in static automation. Consequently, adaptive automation was introduced that required the human operator to perform small manual tasks from time to time.

Rouse [66] proposed two cornerstones for an adaptive aiding system: human performance monitoring and online assessment methods. Both these components are humancentric (i.e. humans must monitor and evaluate the current state of task demands and then compare this state with an estimate of the human information processing resources and human sensorimotor resources available). Online assessment methods augment the prediction process of human performances by providing information on what the human is doing and intends to do.

Byrne and Parasuraman [67] viewed adaptive automation (i.e. adaptive aiding or adaptive function allocation) as an automation design methodology that dynamically distributes tasks between computer systems and human operators. They explained that psychophysiology can provide information on the effect of available automation forms to enhance the associated adaptive logic, take measurements of the human operator, and integrate these measurements with models of the operator and performance measures to improve the method by which automation is regulated.

The division of labour (i.e. the split between the human and the machine) can be dynamically adjusted based on a multitude of factors, including the skill levels of the operator, the demands of the task, and any system-specific requirements that promote optimal performance. A human may be delegated a task, not necessarily because the human will be better than the machine at performing the task, but because the assignment will balance the human’s cognitive load while not degrading the overall performance within the environment.

Research identified the following three approaches to generate criteria for adapting automation to the user [66, 68]:

1. Automation that continuously listens to critical events (i.e. environment stimuli) and engages as necessary.
2. In a model-based approach, a priori model of optimal operator performance could be used to schedule automation.
3. An automation that continuously measures operator function (i.e. performance) and mental (i.e. physiological) state.

Parasuraman et al. [68] contended that a hybrid of the above criteria is needed to design robust adaptive systems.

Augmented cognition (AC) [69] has evolved as a form of adaptive automation, whereby both the user and the automation are tightly coupled via physiological and neurological sensors. AC has three components: cognitive state sensors, adaptation strategies, and control systems. The continuous monitoring of the task, electroencephalograph (EEG), and the environment enables real-time validation of the implementation of an AC system [70, 71].

### On CoCyS

The study of human factors and cognitive science has unified the level of abstraction within which humans and machines can be analysed using similar methods and philosophies. Every agent, be it a human or a machine, can receive information, process information, and produce an action. Thus, the relationship between any two thinking entities can be analysed and combined from the perspective of information processing and decision-making. This level of abstraction is referred to as information–processing–action (IPA). The term IPS has been used to emphasise the information and decision-making aspect, and the phrase sensors–processors–actuators (SPA) has been used to emphasise the platform or mediums through which the interaction lives and takes place.

**Fig. 6:**
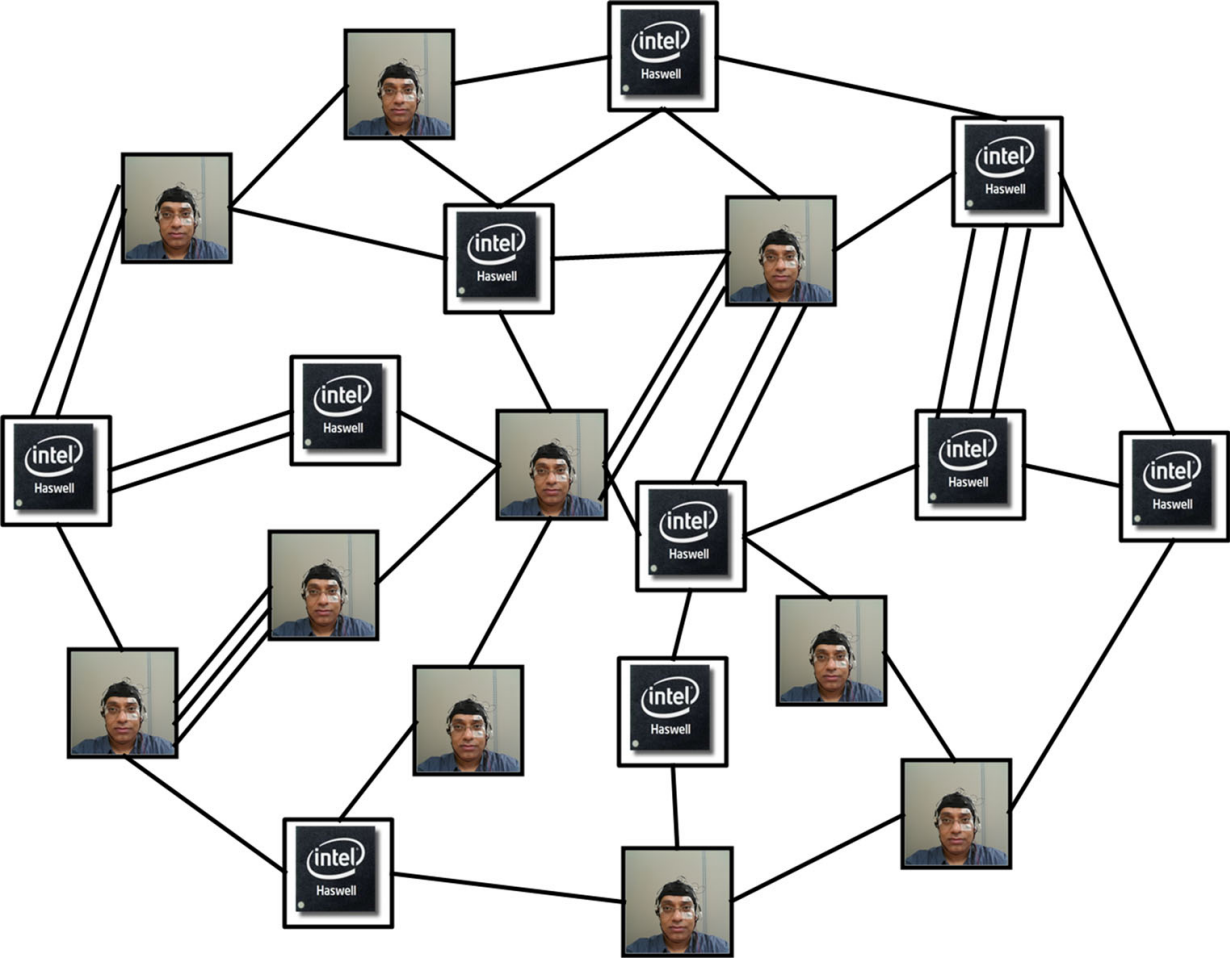
A pictorial representation of CoCyS as a cloud made of humans and machines

CoCyS characterises the interaction among entities as a multimode network (i.e. multiple links can exist between two nodes that depict different modes or relationships) (see Fig. 6) and thus extends previous literature in the following ways:

1. Nodes represent entities, and each link represents a relationship. Thus, each link in CoCyS represents a unique context for interaction; for example, an air traffic controller interacts with automation to form a situation awareness picture of the traffic. An IPA or SPA approach to a system makes context crucial; if the type of relationship changes, the context changes. Thus, CoCyS emphasises the need for an explicit representation of contexts.
  Each node in CoCyS may have multiple connections to other nodes and may connect to more than one other node; thus, all links connected to a node represent all the contexts within which an agent lives in the overall network. Accordingly, a node represents an agent, but also acts as an integration point for all the contexts within which an agent is embodied and situated.
2. CoCyS emphasises the similarities between cyber space (i.e. information flows in an electromagnetic spectrum) and cognitive space (i.e. information flows in brain electromagnetic signals). However, it does not limit the interaction between humans and machines to classic signal processing; rather, it emphasises these similarities to demonstrate that interactions must occur in a natural space occupied by humans and machines in a particular context. Thus, CoCyS emphasises the need to find a natural space within which to model a type of interaction.
  The implications of the above discussion are critical. A human speaks to another human (in the same language) to negotiate contexts. A machine transfers data to another machine in a common format. Thus, human–human and machine–machine interactions live in a natural space. However, when humans interact with machines, this natural space ceases to exist. Previously, humans used a mouse to move a cursor on a screen, now humans use their fingers on a touch screen to move the cursor. The relationship has become more natural for the human (i.e. closer to human–human interaction), but is the space natural? Can a computer reply by sending signals to hands that a human can understand? Steps have been taken to place the interaction in natural space; however, the space is not yet sufficiently natural.
  An example of a natural space is natural language processing, where humans and machines talk to each other naturally. A robot that uses its hand to shake a human hand or exerts pressure to show passion or confidence is an example of a type of interaction that occurs in a natural space.
  These two examples emphasise that for humans and machines to interact in a natural space, each interaction must have an IPA and SPA lens. An interaction is a flow of information between two agents occurring within a physical medium.
3. CoCyS takes the view that human–machine teaming is a cloud (i.e. a form of cloud computing) in which each human is seen as a computational machine. The view is more than a metaphor; it emphasises a number of issues that are pertinent to the success of human–machine teaming including:
  We can attempt to model intangibles such as trust and emotions when morphing humans and machines; however, viewing CoCyS as a cloud shows that any model needs to be computational. Feelings and intangibles are mechanistic and cannot equate to human understandings of these concepts.
  Examining augmentation in CoCyS reveals that augmentation is a fusion of different computational agents; a computational model is run by a machine and a human. This may sound philosophical; however, it is essential that any ambiguity in analysing or understanding the augmentation process is removed.
  Humans and machines in CoCyS are seen as software on the application layer of a cloud. Software does not need to know anything about the underlying hardware infrastructure (this important point will be revisited later in this paper).
4. CoCyS is a network of autonomous human and machine agents. Links represent relationships. If agents are allowed to communicate, communication links appear in CoCyS. The absence of communication links represents cases where agents are not allowed to communicate. Autonomy plays a key role in this particular case, where agents should still be successful in completing their goals despite the lack of communication.

Given the above, we define CoCyS as:

#### **Definition 14**

CoCyS is a cloud computing environment with the nodes in the cloud representing humans and machines.

#### **Definition 15**

CoCyS is a network of computational machines that communicate with each other smoothly, seamlessly and naturally.

### The Architecture of a Cookie

**Fig. 7:**
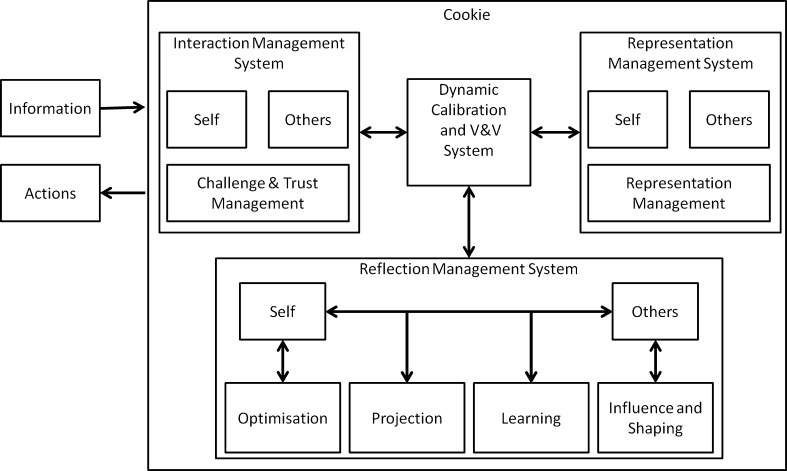
Architecture of a Cookie

A Cookie (see Fig. 7) is the name we give to a TA node/agent in CoCyS. Each Cookie is a computational environment that manages the relationship between any human–human, human–machine or machine–machine CoCyS pairs of agents. A Cookie has the same architecture regardless of whether it is interfacing with a human or a machine. For a human, this architecture represents the protocol for training the human to think and act within an environment. All Cookies in a network have the same architecture, but may change in terms of data and models dependent upon the contexts established by a link. Figure 7 shows the four systems of a Cookie.

- *Representation Management System* This system is responsible for representing self, others and managing these representations.
  A Cookie needs an internal representation to represent itself. Philosophically, it is not being suggested that a Cookie needs to be self-conscious; however, a Cookie needs to have a representation that enables it to learn and optimise its performance.
  The environment of a Cookie is made up of all other interacting Cookies. Thus, for a Cookie to interact smartly with other Cookies, it needs to have a representation for any other Cookie with which it interacts.
  A Cookie also needs to be able to manage its internal representations and adapt these representations to meet environmental demands. Adapting internal representations is one of the main challenges in TA and CoCyS and is discussed further below.
- *Reflection Management System* A Cookie needs to continuously learn about itself as it interacts with the environment. Self-learning is a crucial feedback mechanism for successful interactions. By learning about itself, a cookie can predict future actions and select an appropriate action. Further, to optimise its own actions, a Cookie should optimise its own representation and learn to self-process.
- *Projecting, influencing, and shaping others* A Cookie needs to learn about other Cookies or agents. Such learning will allow the Cookie to project actions in the future and anticipate the likely actions of other Cookies. A Cookie cannot optimise other Cookies, but can identify the best actions to influence and shape other Cookies in the environment.
- *Interaction Management System* Learning, projection, and optimisation of self are three classic components that do not allow for the emergence of innovation and novelty. When a Cookie challenges itself, it is able to go beyond the norm and extend its own abilities, and when it challenges other Cookies in its environment, it is able to shape the environment in a beneficial direction. Challenge and trust need to be considered together; trust is influenced by an agent’s actions, and challenge is the process of pushing an agent beyond its performance envelope or comfort zone. Thus, these two concepts are rooted in similar principles.
- *Dynamic Calibration System* A major problem of real-time systems is that the continuously evolving nature of software requires a continuous ability to validate the system and calibrate internal models. A self-calibration and validation process changes the system from one that depends on a human engineer for maintenance to a system that can self-maintain.

## Open Challenges for TA and CoCyS

Advances in artificial intelligence, engineering, and technologies continue to increase exponentially. However, there are many fundamental problems that need to be resolved before TA and CoCyS can be realised. Today, simple CoCyS systems exist on a small scale (i.e. between one human and one machine or a small group of humans and machines), but if some of the fundamental challenges of CoCyS are not addressed, true smartness and intelligence in human–machine interactions will continue to be limited.

- *Autonomous Context Representation* Each link in CoCyS is a type of relationship between two agents that live within an environment known as context. This raises two fundamental research questions: How can a new context be detected? And How can a new context be represented efficiently?
  A relationship can be viewed as system boundary on a context; for example, motherhood defines the context within which a child interacts with his/her mother. If a mother is also the manager of a business where the child works, a new link is established that defines a different relationship between the two agents and a new context.
  Each Cookie must have the autonomous ability to represent a new context. However, a representation is not just a simple data structure that holds information about a context, it also encodes the boundary constraints that define what the agent can and cannot do within the context, the objective of the relationship and the value system within which it operates.
  To represent contexts autonomously, the agent needs to be able to act deliberately on the environment and self-generate a series of goal-oriented queries that enable it to acquire the necessary information to represent the new context.
- *Automatically Defined Indicators* Indicators are the magnets of any process search for solutions. Without indicators, the objective is unknowable. A Cookie will encounter many contexts that differ in their form and nature to contexts tested during design. Thus, a Cookie must be able to automatically define new indicators for guidance in these contexts. This raises the following research questions: What is the relationship between contexts and indicators? How can this relationship be formally defined to be computable? How can these indicators be automatically synthesised? and How can the interdependency, causal loops, and positive and negative correlations among a set of indicators be automatically inferred?
  These questions are presently too difficult for humans to answer. However, the answers do not need to be optimal; rather, the answers only have to be sufficiently meaningful to ensure that a Cookie is able to adapt and manage unknown environments and contexts. Contexts define the boundary constraints of a situation. A Cookie needs to understand the context and autonomously define its role within a particular context, be able to design an appropriate set of indicators to measure the success of its role, and identify the effect of its actions on its own objectives and understand any other indicators used to measure its performance.
- *Automatically Defined Transformations* The challenge of automatically defining transformations exists within every Cookie system. It raises the following three fundamental research questions: How can a Cookie detect when it is beneficial to transform a representation or to model another? How can an appropriate transformation be discovered? And How can the transformation be performed? A Cookie must be able to transform one representation to another, one model to another, and one piece of information to another; for example, an agent might quickly learn how to play a game using an artificial neural network. This model and type of representation allows for nonlinear relationships to be learnt in a more compact form than classic rule-based systems. However, it is not possible to use a classic neural network to justify an action, as the agent must be able to re-encode the learnt artificial neural network into another representation (e.g. a decision tree or propositional logic) that will enable the agent to reason in relation to its actions [72]. Different situations will necessitate different representations and models. However, having a single form of representation for all models will not only constrain an agent’s ability to learn, it will also limit the efficiency of learning, acting, and reasoning. An agent needs to be equipped with appropriate mechanisms to automatically define a transformation that can be then used to transform one form of its internal models and representations to another.
- *Adaptive Representation of Self* An adaptive representation of self is a necessary step towards developing a Cookie’s ability to reflect on its own actions and think about itself. This challenge raises a number of research questions including What is an appropriate level of abstraction for self? What is an appropriate representation of self? What measurements should a Cookie take to represent itself? and How can a Cookie adapt its representation of self as it evolves within its environment?
  These questions are more related to engineering than philosophy, as the objective of CoCyS was to engineer a Cookie. The question of what self is depends on context; self represents the role of a Cookie in its interaction in some contexts. Thus, the level of abstraction to represent self must be appropriate to achieve the objective of the interactions in different contexts. Once a Cookie makes an abstract decision, representation and measurement questions follow. The adaptation question raises additional challenges, as a Cookie accumulates experiences through different interactions; thus, original levels of abstraction, representation, and measurements must be continuously revisited. This is a challenging task that requires an agent to be able to change its self-representation and the performance metrics required to self-evaluate.
- *Autonomous Association* CoCyS creates a collaborative environment. If two agents are competing, the competition works within a collaborative framework on a systems level. Thus, the underlying objective of CoCyS was collaborative in nature. Autonomous association is a core fundamental mechanism that underpins cooperative interactions. Two agents will act together to cooperate if they see that a cooperative relationship will enable them to achieve their goals better than working in isolation. Three fundamental research questions arise: Under what conditions does an agent decide that an association with one or more agents is necessary? How can an agent observe an opportunity for cooperation in an environment that creates more benefits for the agent? and What are the mechanisms and operators with which an agent needs to be equipped for this association to occur autonomously?
  All three questions are of a great significance to an agent’s ability to enter into a cooperative relationship. To answer the first question, an agent needs to understand its own context, abilities and goals to make decisions to seek associations with one or more other agents. The second question requires that an agent continuously evaluates every interaction in its environment and seeks opportunities to leverage these interactions for other purposes. The third question focuses on the planning and communication abilities of an agent to engage with others.
- *Smooth Interaction* The success in implementing a symbiotic process can be measured by the level of ease with which the interaction occurs. It is asserted that within interactions, there are two broad categories of complexities, that is, complexity of communication and complexity of negotiation. The first type of complexity depends on how advanced a Cookie is in reciprocating the conversation with and relative to the other connected nodes/Cookies. The second type depends on the advancement of a Cookie’s internal decision-making process. A smooth interaction is associated with the first complexity.
  This raises a number of challenging research questions including What is an appropriate set of interfaces for communication in different contexts and between different parties? What is an adequate medium for communication in a Cookie? What is the relationship between the appropriateness of the communication language and the context of a Cookie’s interaction?
  These questions are difficult, as a clear mechanism needs to exist to allow a Cookie to choose an appropriate answer to each question. A Cookie must be able to optimise the answer to each question based on the context in which and the parties with whom it interacts.
- *Deciding and Reasoning* A major challenge in the design of a Cookie is that algorithms must produce autonomous actions and reasoning . An autonomous agent needs to autonomously make decisions. However, in a time-constrained environment, the agent needs to quickly make decisions and produce actions. Further, the agent must be able to reason in relation to its actions if asked. This raises a number of research questions including: How can the need for fast action production be balanced against the need for reasoning that requires an agent to logically infer actions from knowledge and premises? and Can action production be separated from reasoning?
  The last question in particularly is important in TAS and CoCyS. Mathematical models such as feed forward artificial neural networks can be relied on to produce actions; however, while these models are fast, they are black boxes with limited capacities to reason about the actions produced. Conversely, classic reasoning (that uses some form of logic) is known to be slow and does not scale well in complex environments. Equally important, as a knowledge base grows, it becomes difficult to maintain the symbolic knowledge inside the knowledge base and keep this knowledge up to date.
  This suggests that action production should be separated from reasoning (see [73, 74] for an example). An action can be generated using an artificial neural network, and when questioned about the action, classic reasoning can be used based on a symbolic knowledge base. To ensure that the two models are consistent in their behaviours, it is important that forms of rule extraction from the neural network are used to construct a knowledge base [72] and maximise consistency and compatibility.
- *Uncertainty Management* Uncertainty is a major concern in the design of a Cookie and may occur for a variety of reasons, including due to a Cookie’s limited knowledge of another Cookie or an environment, the levels of abstraction and fidelity a Cookie employs in one or more internal models, the deliberate deceptive actions from other Cookies or the nature of the context within which it is embedded. Uncertainty management raises a number of research questions including: How can a Cookie be designed to be aware of uncertainty? What are the operators and mechanisms needed to smartly manage uncertainty in interactions? and How should we model uncertainty (and prevent models from being subject to uncertainty) [75]?
  The main difference between models designed to manage uncertainty and those not designed to manage uncertainty lies in the ability of the model to adapt its structure and level of abstraction. In classic modelling design, a model has parameters and variables, and all uncertainties are known in advance. However, this design is inappropriate for a Cookie encountering novel uncertainties. A Cookie needs to adapt the internal structure of its model or even change its own model to manage new uncertainties in the environment. A prerequisite for this process is that a Cookie must be able to recognise uncertainty, as it is only when this occurs that a Cookie can initiate the process to adapt and change its model.
- *Autonomous Analytics* The discussion on uncertainty also demonstrates that a Cookie must be equipped with the ability to autonomously perform data and decision analyses. A Cookie must have the ability to engage in a process of data analytics in different environments to identify the objective of the exercise autonomously, identify what data need to be collected and from where, design a plan for data collection, decide which models are appropriate for learning, collect the data, train the model, validate the model, and finally, add the model to its model base. Important research questions associated with this challenge include: How can the objective of an analytic exercise be autonomously defined? How can the appropriate level of abstraction, resolution, fidelity, and model for a problem be autonomously chosen? How can a Cookie autonomously decide on the appropriateness of a data set? How can a Cookie autonomously fix a data set deemed inappropriate? and How can a Cookie autonomously validate a model?
  The above questions are the important in autonomous analytics. They highlight some of the questions currently being debated by analysts and the level of challenges faced in automating the challenges. However, progress can be made in relation to each of these questions by considering existing technologies and architectures that may be used to achieve this progress (see [76] for more details). The underlying key challenge for each of these questions is the need for the analytical software to be context-aware, self-regulate its decisions within a context and be able to evaluate its analytics’ decisions within a context.
- *Trust Analytics* We mentioned a number of situations in which an understanding of trust needs to be embedded within each party involved in the trusting decision. Trust necessitates that each party assumes responsibility for some level of risk. To accept this responsibility, a party needs to be able to assess the risk in the situation. The term trust analytics refers to the data and decision analyses that each party must undertake to assess, make, and invoke decisions on trust. A number of research questions arise including: How can trust be modelled as a conscious process by which an agent blends its emotions, experiences, and knowledge to make decisions as a truster or trustee? How can a trusting decision be autonomously evaluated? and How should a Cookie autonomously present trusted results to a decision-maker?
  These questions focus on the trusting decision. To answer these questions, mechanisms for integrating the experience of an agent must be designed. Any such integration process must be efficient. Agents need to be able to combine multimodal information such as emotions, knowledge, and skills, to make a trusting decisions. When a human is a party, the Cookie must identify the best method to communicate to the human. If the best method is through visualisation , the background knowledge of the human needs to be analysed to estimate the most appropriate information for visualisation and the way in which this information can be visualised; for example, if the human is a network administrator, a Cookie needs to visualise the information for a specialist with the human’s level of skills and expertise.
- *Influence and Shaping Operators* As stated above, an agent needs to be an active participant in a trusting relationship (e.g. if a truster or trustee perceives that another party should not be trusted or is not sufficiently trustworthy, an agent must be active in influencing the other agent or shaping the environment to change this perception). The operators of influence and shaping can be used to achieve this result. A number of research questions arise including: How can an agent be autonomously influenced or an environment shaped by designing and monitoring the impact of actions on another agent or the environment? How can key constraints and indicators be identified to target influencing and shaping operations? and How can trust be influenced and shaped?
  The above questions should be relevant to any decisions a Cookie makes. Decision should not be made in a vacuum. Decisions are made for purposes (i.e. to influence another entity or the environment) to achieve an effect (i.e. to maximise the benefits/objectives of an agent). Thus, a Cookie needs to autonomously design actions in the light of purposes and effects and this is where Computational Red Teaming (CRT) has an important role.
- *Computational Red Teaming* (*CRT*) Any conscious negotiation between two humans appears to involve the autonomous calibration of the mental models of one human and another and the context of the interaction. Simply put, an educated negotiation may be based on the following thought process: ‘If I propose *X*1, he/she might propose *Y*2 or *Y*3, but if I propose *X*2, he/she might propose *Y*4 or *Y*5; thus, I will propose *X*1 because *Y*2 and *Y*3 appear better on average’. If this choice and reasoning process is undertaken by thinking for the other person (rather than merely anticipating their actions from the outside), it is called ‘red teaming’. When it is done systematically by humans or in silico, it is called CRT. The previous example should not be confused with classic reasoning because it relies on more complex forms of decision-making and reasoning models. Simulation, optimisation, and data mining using computational intelligence techniques, when combined, offer powerful methodologies for CRT. Many research questions arise in relation to CRT, and readers are referred to [76] for a detailed discussion on the topic.
- *Continuous Calibration* No model remains accurate indefinitely. Even if the structure of a model does not need to change, the parameters of a model may need to change. Research questions about continuous calibration include: How can a baseline be autonomously defined for calibration? How can events be autonomously selected in a naturalistic setting for calibration? and What metrics should be used to assess a successful calibration under different environmental and uncertain conditions?
  The above questions must be answered before any real-time autonomous system is able to work continuously in different environments; for example, when analysing EEG data, variations may exist between subjects, within the same subject at different times of day, in different contexts, and in different environments. Fixing the model to extract features from the EEG would very quickly make the model inappropriate. Continuous autonomous calibration enables the model to adapt its parameters or even its structure in different settings without human interference.
- *ContinuousV*&*V* A smart human continuously and consciously validates and verifies his/her understanding of his/her surrounding environment. This is very different from classic software engineering approaches, where an assumption exists that a model will be verified and validated as used. If the complexity of the software were to be embedded within a serious sophisticated autonomous agent or a Cookie, the classic cycle is no longer feasible, as validation needs to be performed continuously. This raises a number of research questions, including: How can validation and verification (V&V) be continuously assessed? If V&V becomes another piece of software, does the V&V process itself need to undergo V&V? and How can the embedded continuous V&V process be used to discover problems and determine responses to these problems?
  The above questions seem to form a vicious cycle. This cycle can only be broken when it is accepted that (like humans) machines will make mistakes, and all we can do is ensure that these mistakes are not catastrophic. There are different ways to reduce the chances of mistakes becoming catastrophic (e.g. designing cars to crumple in a crash to absorb the energy from the crash and reduce risks to passengers is an example of the form (i.e. body) and matter (i.e. material) of cars being designed for safety, even if the software of the autonomous car fails in the crash). Interestingly, this example shows that V&V is similar to risk mitigation and that an overlapping multilayered approach must be taken to achieve protections across systems.

## Future Work

**Fig. 8:**
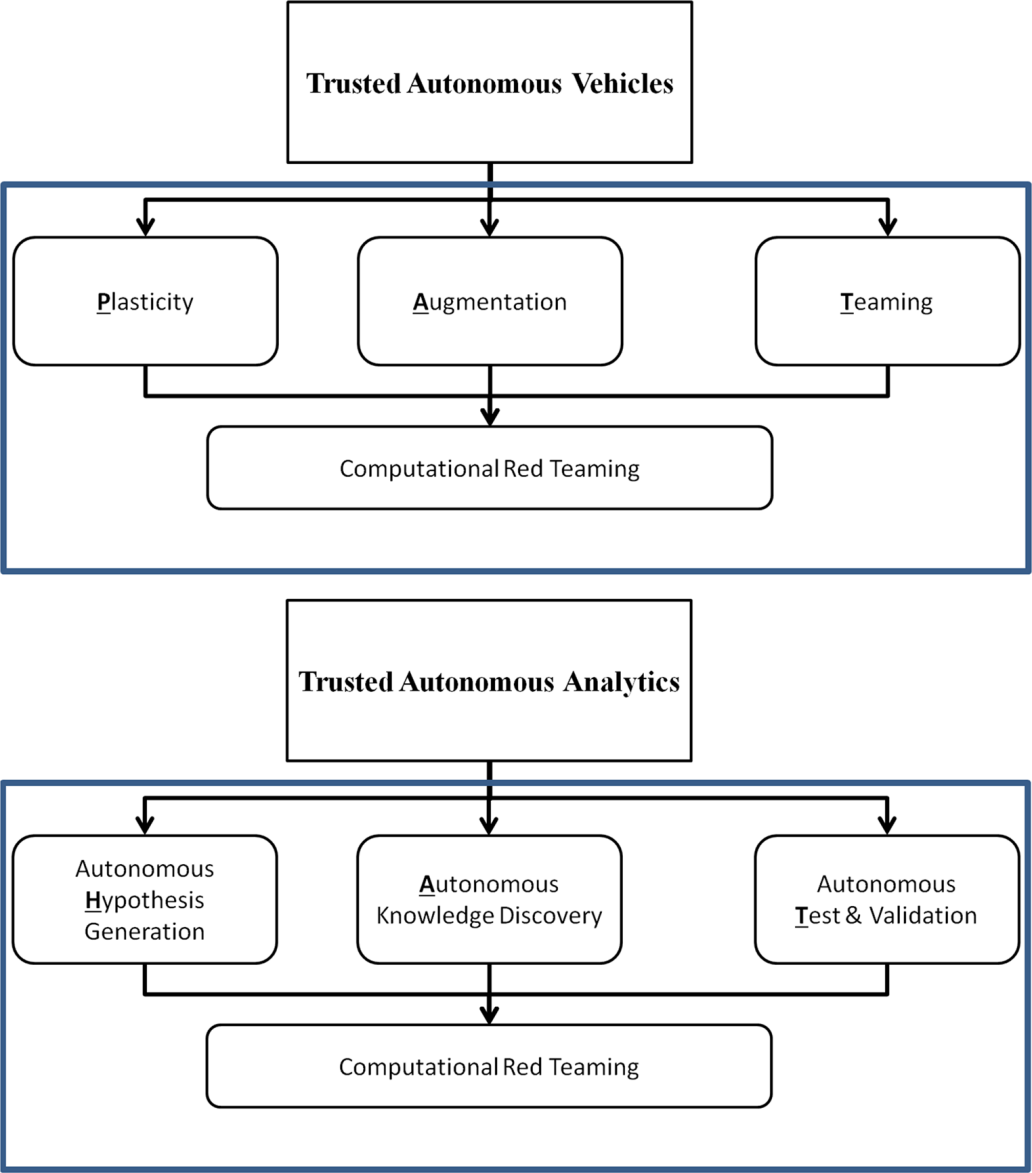
Functional view of trusted autonomous vehicles and trusted autonomous analytics

Figure 8 shows the two sides of the TA coin. The first side represents hard-core engineering, whereby autonomous systems are seen as physical pieces of hardware known as robots or vehicles. The second side takes the approach that autonomy exists in software; thus, a TAS needs to be equipped with the necessary analytical tools and techniques to exercise autonomy.

To design a TAS, four broad issues must be addressed. First, the issue of plasticity, that is, a system must be able to continue to operate robustly and adapt itself to an unanticipated environment. Plasticity as an internal characteristic of a vehicle is not sufficient. The system or the robot needs to be able to augment itself with any sensors available in its environment to maximise its own situation awareness and benefits. This process requires self-consciousness so that the vehicle can identify its own vulnerabilities and leverage elements available in the environment to its own advantage.

The third issue is that the system must be able to team with others as necessary. This issue emphasises the ability of the vehicle to interface with others. The fourth issue relates to ‘red teaming’ [77], that is, a system needs to play devil’s advocate with itself and have the ability to self-evaluate.

Analytics view TASs from a software perspective and requires that the system be able to generate its own hypothesis, evaluate this hypothesis, and be able to self-judge and self-test.

In the light of above, the pictorial equation of TA was designed as:

$$\text {Trusted}\; \text{Autonomy} = \text {PAT} + \text {HAT}$$

Future work on TAS will largely touch on one or more aspects of the previous equation. However, only when all components of the equation are addressed, TAS will be both trusted and autonomous.

## Conclusion

In this paper, we presented an overview of the literature on trust and autonomy. TA was considered in the light of previous research, and relevant definitions and conceptualisations were provided. The concept of a trusted autonomous agent was then generalised to a network made of humans and machines that use CoCyS. The architecture of a node in CoCyS (i.e. a Cookie) was presented in sufficient detail to map the main ingredients of the concept. The paper then concluded by discussing open challenges in the areas of TA and CoCyS.
